# Changing Host Plants Causes Structural Differences in the Parasitoid Complex of the Monophagous Moth *Yponomeuta evonymella*, but Does Not Improve Survival Rate

**DOI:** 10.3390/insects10070197

**Published:** 2019-07-04

**Authors:** Adrian Łukowski, Wanda Janek, Edward Baraniak, Urszula Walczak, Piotr Karolewski

**Affiliations:** 1 Faculty of Forestry, Poznań University of Life Sciences, Wojska Polskiego 71c, 60-625 Poznań, Poland; 2 Laboratory of Ecology, Institute of Dendrology Polish Academy of Sciences, Parkowa 5, 62-035 Kórnik, Poland; 3 Faculty of Biology, Adam Mickiewicz University, Umultowska 89, 61-614 Poznań, Poland

**Keywords:** biological control, ermine moth, enemy-free space, food base, folivorous insect, Lepidoptera, native and invasive species, *Prunus padus*, *P. serotina*, *Yponomeuta evonymella*, Yponomeutidae

## Abstract

Recently in Poland, cases of host expansion have frequently been observed in the typically monophagous bird-cherry ermine moth (*Yponomeuta evonymella*), which has moved from its native host plant, bird cherry (*Prunus padus*), to a new, widely distributed plant that is invasive in Europe, black cherry (*P. serotina*). We attempted to verify the reasons behind this host change in the context of the enemy-free space hypothesis by focusing on parasitoids attacking larval *Y. evonymella* on one of three host plant variants: The primary host, *P. padus*; initially *P. padus* and later *P. serotina* (*P. padus*/*P. serotina*); or the new host, *P. serotina*. This experiment investigated if changing the host plant could be beneficial to *Y. evonymella* in terms of escaping from harmful parasitoids and improving survival rate. We identified nine species of parasitoids that attack larval *Y. evonymella*, and we found that the number of parasitoid species showed a downward trend from the primary host plant to the *P. padus*/*P. serotina* combination to the new host plant alone. We observed a significant difference among variants in relation to the percentage of cocoons killed by specific parasitoids, but no effects of non-specific parasitoids or other factors. Total mortality did not significantly differ (ca. 37%) among larval rearing variants. Changing the host plant caused differences in the structure of the parasitoid complex of *Y. evonymella* but did not improve its survival rate. This study does not indicate that the host expansion of *Y. evonymella* is associated with the enemy-free space hypothesis; we therefore discuss alternative scenarios that may be more likely.

## 1. Introduction

Plants and the herbivores that feed on their tissues and sap have coevolved, creating a relationship wherein plants produce new defences and herbivores attempt to mitigate those defences to further feed on the plants. Generalists, which include polyphagous and oligophagous insects, feed on a wide range of hosts because they are not specialised to feed on a particular range of plants, whereas specialists are insects that are monophagous. When choosing their host, monophagous insects search for the best food quality and the best environment in terms of the survival of their offspring (see preference-performance hypothesis). An insect’s choice of host plant depends not only on the insect’s food preferences but also on its ability to use the host (e.g., the possibility of actively moving to other plant species). The situation therefore depends on the chemical composition of the new host plant as well as the availability of the host species in the insect’s habitat [1,2,3]. Under conditions where the optimal host is a rare species or there is heavy competition for food, even monophagous insects can be forced to use a new plant species [3,4]. In cases of invasions by new plant species, a potential host becomes available that was not available before, which may also mean that there is now an open niche, as suggested by the open-niche hypothesis [5]. Alternatively, the enemy-free space hypothesis states that insects will use new host plants to escape from natural enemies [6]. A decrease in the number of natural enemies is usually observed in the case of an insect species relocating [7], but in the long term, parasitoid, and predator species are also able to utilise new herbivorous species [8]. Choosing a new host plant can result in a decrease in adult insect mass [9]; this may extend larval development time, thereby increasing larval mortality due to prolonged exposure to predators and parasitoids [10,11,12]. Some studies have shown, however, that insects feeding on alien plant species, especially those that feed on congeneric alien plant species, develop as well as or better than they would if feeding on native host plants [13]. The reason for this may be that native host plants defend more effectively against coevolved herbivores than do alien plants [14,15,16]. Other studies have shown that many insects cannot successfully switch to introduced congeners [17]. Searching for an alternative food source is a gradual process and insects are most likely to utilise the most abundant host species [2]. This appears to favour the colonisation of invasive species, characterised by mass occurrence.

In this study, we focused on the bird-cherry ermine moth *Yponomeuta evonymella* (L.) (Lepidoptera; Yponomeutidae), which feeds on the European bird cherry (*Prunus padus* L.) and, recently, also on the non-native, invasive black cherry (*P. serotina* Ehrh.). *Y. evonymella* is a moth native to the temperate zone in Europe, Siberia, India, and China. In Europe, it is the most common lepidopteran species that feeds on *P. padus* leaves [18,19,20,21]. In our previous study, we found that the leaves of both black and bird cherry are good quality foods for *Y. evonymella*, when considering mass and survival of insects, concentrations of defensive compounds, and the toughness of leaves [22]. Larvae were observed to feed on the leaves of *P. serotina* shrubs when they were in close proximity to branches of *P. padus* [22]. Larval movement from *P. padus* (where they hatch) onto *P. serotina* during the early stages of larval development is often due to extensive defoliation of *P. padus* shrubs. Recently, we have observed more and more *Y. evonymella* egg clusters being oviposited on the stems of *P. serotina* [23]. 

In European forests, plant species of the family Rosaceae, including the genus *Prunus* L., play a key role in the species composition of the understory [24]. Particularly widespread are shrubs or, more rarely, trees of *P. padus* and the closely related *P. serotina*. Bird cherry is a species native to Europe which has a wide geographic range that extends from the southern to the northern limits of the continent [25,26]. In contrast, black cherry is an alien species to Europe, originating from the north-eastern and central parts of the USA and Mexico, as well as the north of South America [24]. Both species are found in similar natural habitats in Poland [27]; however, black cherry can be observed growing in soil that is extremely poor in minerals or in heavily anthropogenically transformed habitats. Both species are therefore good model systems for studying the interaction of native and non-native plant species from the perspective of native insects changing host plants. 

The leaves of *P. padus*, which begin to appear very early (earlier than *P. serotina* leaves), are heavily attacked by herbivores. This shrub is a suitable source of food for 28 herbivorous insect species, four of which are considered to be host specific [19]. The most abundant of these insects, *Gonioctena quinquepunctata* (F.), *Rhopalosiphum padi* (L.), and *Y. evonymella*, attack *P. padus* every year and in great abundance, causing meaningful damage by consuming half of the total amount of a shrub’s leaves or even causing total defoliation [21,28]. Every ten years, the number of *Y. evonymella* individuals increases, and, every twenty years, gradations appear [18]. Populations of *Y. evonymella* are substantially reduced by natural enemies, such as bird predators and parasitoids. Historically in Europe, 34 parasitoids and 15 predators of *Y. evonymella* have been reported [29]. A recently updated checklist of the diversity of natural enemies of European species of the genus *Yponomeuta* provides information concerning 60 species [30]. The main parasitoids of *Y. evonymella* in Europe are *Ageniaspis fuscicollis* Dalman (Hymenoptera; Encyrtidae), and *Diadegma armillata* Grav. (Hymenoptera; Ichneumonidae), whereas in Korea the most important parasitoid species are *D. armillata*, *Herpestomus brunnicornis* Grav. (Hymenoptera; Ichneumonidae), and *Zenillia dolosa* Meig. (Diptera; Tachinidae) [29].

In this study, we investigated the parasitoid complex of *Y. evonymella* in light of its recently discovered colonisation of a new host plant. The aim of this study was to determine if larval stage-specific parasitism is the main reason for the observed change of host plant exhibited by *Y. evonymella.* Employing an experiment based on three variants of larval feeding—on the primary host *P. padus*, partly on *P. padus* and later on *P. serotina* (*P. padus*/*P. serotina*), or on the new host *P. serotina*—we attempted to explain this larval behaviour by posing a question: Is the observed host expansion of *Y. evonymella* associated with the enemy-free space hypothesis—does *Y. evonymella* feed on *P. serotina* to avoid parasitoids? 

## 2. Materials and Methods 

### 2.1. Insects

This study was carried out on a herbivorous insect, the bird-cherry ermine moth *Y. evonymella* (L.) (Lepidoptera; Yponomeutidae), which primarily feeds on bird cherry *P. padus* L. [19] but is lately more often found on invasive non-native black cherry *P. serotina* Ehrh. [22]. During this study, we used larvae, pupae, and adults.

### 2.2. Site and Plant Material

Larval stage-specific parasitism of *Y. evonymella* was studied in Poland in 2014 in Kobylepole Forest (Babki Forest District; 52°38’61’’ N and 17°04’54’’ E). The research was carried out on two species of undergrowth shrubs: Black cherry (*P. serotina* Ehrh.; non-native species in Europe) and bird cherry (*P. padus* L.; native species in Europe). Our previous field investigations conducted in Kobylepole Forest were indicative of the abundant presence of both species of *Prunus* in the forest’s undergrowth layer. Both investigated understory shrubs grow in mid-fertile soil under the canopy of coniferous forests and mixed forests consisting of various tree species (mainly *Pinus sylvestris* L. mixed with *Quercus robur* L.). In the late summer of 2013, 24 typical shrubs (12 of *P. padus* and 12 of *P. serotina*) growing in temperate shade (under the tree canopy; approximately 50% full sunlight) were randomly chosen so that their canopy dimensions, ages, and heights (3–5 m) were similar. 

### 2.3. Study Design

To identify and estimate the number of natural parasitoids of *Y. evonymella* in this field experiment, we used larvae in the L1 stage of development (before their emergence from under the domed refuges covering egg batches), which we collected in the autumn of 2013, because searching for larvae was simpler in the leafless period. Twenty-four shoots (10–15 cm) with egg batches collected from shrubs of *P. padus* growing in Kobylepole Forest were overwintered in external controlled conditions by placing the shoots vertically in a pot filled with sand and covering them with a plastic net that guaranteed good ventilation and protection from insectivorous birds. In the spring, the collected shoots containing egg batches were attached randomly with coated wire to places on shrubs of *P. padus* or *P. serotina* where eggs are usually laid (short lateral shoots), at the time when larvae of this species begin to feed (mid-April 2014). Moreover, during a field inventory process held in Kobylepole Forest (beginning of May 2014), we marked 12 places where we noticed that larval *Y. evonymella* that had originally been grazing on *P. padus* had spontaneously moved to shrubs of *P. serotina* in their close vicinity. In summary, we created three different variants of larval rearing: On the primary host *P. padus*, initially on *P. padus* and later on *P. serotina* (*P. padus*/*P. serotina*), and on the new host *P. serotina* alone.

When the first larvae began to pupate (beginning of June), the pupae and individual larvae of the last stadium (5th instar) were collected and moved to the laboratory. Shoots with aggregates of pupae were cut out of the shrubs and placed separately in one-litre plastic containers. Larvae that did not manage to pupate in the field had access to leaves of the proper shrub species. The vast majority pupated within 24 h, although some larvae did not pupate in the entire time of the experiment, which is a peculiar defence mechanism of this species, as some larvae entwine their cocoons with those of the colony to the end of their life in an effort to protect themselves. As the containers were filled with collected material, we placed them in a room with stable temperature and light conditions (22 °C, day:night cycle of 16 h:8 h). Every container was checked daily to observe and record not only the emergence of mature forms of *Y. evonymella* individuals but also the emergence of parasitoids. Moths that had just emerged were removed the same day, sex was determined, and further tests—irrelevant to the current study—were conducted. The parasitoids that emerged were kept for further identification. We also determined that there were a number of cocoons from which moths did not emerge because of other factors (e.g., a moth being blocked in the process of leaving the pupa, viruses, and pathogens).

When we observed that Dipteran larvae emerged from pupal *Y. evonymella*, these pupae were moved to another separate vessel to avoid accidental infection of healthy pupae. Similarly, when polyembrionic *A. fuscicollis* was observed, pupae with visible marks showing emergence of this parasitoid were taken out of the container, but they were not included in this study because they are egg parasitoids. 

### 2.4. Statistical Analyses

The percentage of parasitism of each parasitoid species was calculated by dividing the number of individual pupae killed by the parasitoid by the number of pupae collected for the host variant in nature. The average parasitism of each parasitoid species was calculated by averaging the percentage of parasitism of each parasitoid species for each host-plant variant. The total mortality was obtained by adding the percentage of parasitism of each parasitoid species to the percentage of pupae killed by other factors. To evaluate the importance of each parasitoid, we identified significant differences in percentage of parasitism using the Kruskal–Wallis Test and Dunn’s Test (post-hoc; *p* < 0.05). The average total parasitism was calculated by adding the percentage of parasitism of each parasitoid. A standard deviation (SD) was given for each mean value. All calculations were performed using JMP Pro 13.0 software (SAS Institute Inc., Cary, NC, USA).

## 3. Results

The structure of the *Y. evonymella* parasitoid complex and the richness of the various species were analysed in three different variants of larval rearing. In this study, we found and identified nine species of parasitoids attacking larval *Y. evonymella* (Table 1). The main group of insects parasitizing *Y. evonymella* are individuals from the order Hymenoptera, which caused more mortality than those from Diptera. We found the following parasitic species: *Bactromyia aurulenta* (Meig.) (Diptera; Tachinidae) and *Baryscapus evonymellae* (Bché.) (Hymenoptera; Eulophidae), as well as six taxa from the family Ichneumonidae (Hymenoptera), including *Agrypon anxium* (Wesm.), *D. armillata* (Grav.), *H. brunnicornis* (Grav.), *Itoplectis maculator* (F.), *Lissonota* sp. Grav., *Dirophanes invisor* (Thunb.), and *Pimpla turionellae* L.

The composition of parasitoid species was more diverse when larvae fed on *P. padus* than when they fed on *P. serotina*. There was a downward trend in the number of parasitoid species present, from the primary host plant variant of larval rearing (*P. padus*) to the partial variant (*P. padus*/*P. serotina*) to the new plant (*P. serotina*). All nine parasitoid species found were present in samples reared on *P. padus*, seven were present when larvae were reared partly on *P. padus* and *P. serotina*, and five were present when larvae were reared on *P. serotina* alone. The total mortality for each larval rearing variant, however, was not significantly different, with ca. 37% mortality among larvae feeding on *P. padus*, 32% among those on the *P. padus*/*P. serotina* variant and 38% among those on the *P. serotina* variant (χ^2^ = 0.42; *p* = 0.81). On larvae reared first on *P. padus* and then *P. serotina*, *B. evonymellae* and *P. turionellae* were absent. In turn, in larval samples reared only on *P. serotina*, *B. aurulenta*, *H. brunnicornis*, *I. maculator,* and *D. invisor* were absent. *Bactromyia aurulenta* and *B. evonymellae* were the most abundant general parasitoid on both larvae raised on only *P. padus* and those raised on a mix of *P. padus*/*P. serotina*, whereas in the case of larvae raised on *P. serotina* alone, the most common parasitoid was *A. anxium* (Table 1). 

**Figure 1:**
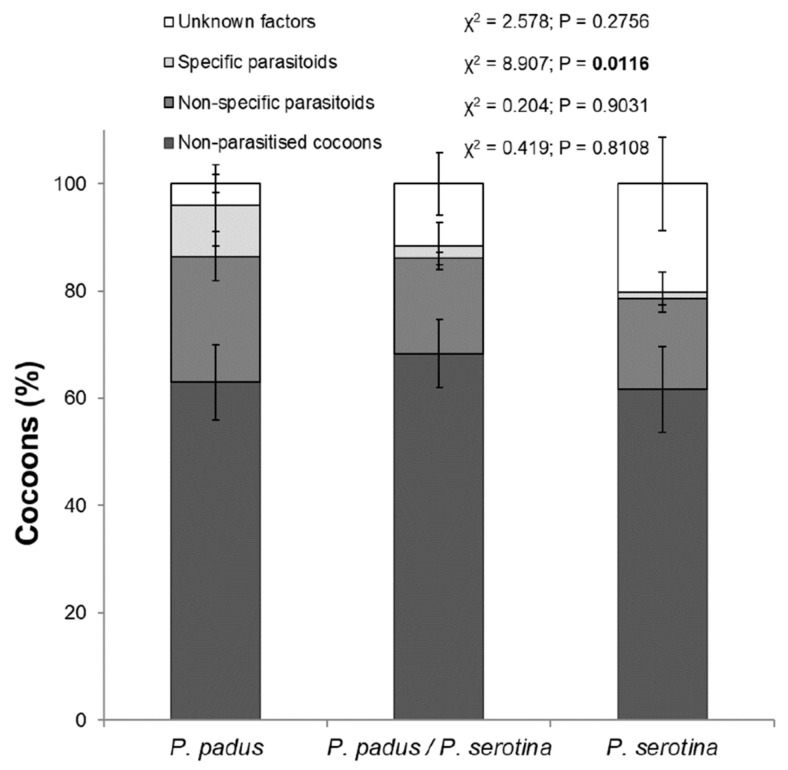
Mortality of pupal *Yponomeuta evonymella* feeding on *Prunus padus*, *P. serotina* or a mix of the two shrub species. Graph shows percentage of collected cocoons (means ± SD) parasitised by specific and non-specific parasitoids, killed by unknown factors, or unparasitised.

The total percentages of parasite-caused mortality (χ^2^ = 2.99; *p* = 0.22) and mortality due to other non-studied factors (e.g., entomopathogenic fungi and damage by predators) for each larval rearing variant were not significantly different (Table 1). Based on the biology and ecology of the studied parasitoids, we divided them into one group of parasites more specific to the genus *Yponomeuta* Latreille (including mono- and oligophagous) and another group of non-specific parasitoid species (polyphagous). The specific parasitoid group included *B. evonymellae* and *H. brunnicornis*. All other parasitic species found were placed in the non-specific group. A closer look at the results (Figure 1) showed a statistically significant difference among feeding variants when only regarding the percentage of cocoons killed by specific parasitoids. In comparison, the percentage of cocoons killed by specific parasitoids in the *P. serotina* variant (“b”; Dunn’s test) was lower than the percentage killed in the *P. padus* alone and the *P. padus*/*P. serotina* variants (both “a”). Furthermore, there were no differences among variants regarding the percentage of cocoons killed by non-specific parasitoids or other factors or regarding the number of non-parasitised cocoons. 

## 4. Discussion

### 4.1. Host Plant Changes Exhibited by *Y. evonymella*

In our study, we investigated the connections among a host plant, the monophagous bird-cherry ermine moth (*Y. evonymella*), and the composition of the moth’s parasitoid complex, depending on the larval–host plant variant. We were searching for an explanation of a phenomenon that has been observed in nature over the past ten years: *Y. evonymella* more and more frequently utilising a new plant, *P. serotina* [22], a non-native and invasive species [24,31]. Generally, *Y. evonymella* hatches and feeds on *P. padus*, but it often also moves to the shoots of *P. serotina* shrubs, where it may then feed and pupate. A recent study confirmed that *Y. evonymella* egg clusters are also observed on the stems of *P. serotina* [23]. In the current study, we determined that utilising this new host does not benefit *Y. evonymella* in terms of reduced larval stage-specific parasitism. Thus, our study does not support our prediction that larval *Y. evonymella* feed on *P. serotina* solely to avoid parasitoids and predators. In a broader sense, the observed host expansion of *Y. evonymella* does not appear to be associated with the enemy-free space hypothesis [7]. Most likely, the main benefit for *Y. evonymella* is simply the expansion of its food base.

As insects are strongly dependent on their preferred food [32,33], feeding on the optimal host plant allows them to obtain a larger body size [34]. Choosing a suboptimal host plant could result in a reduction in body mass [3,9,35] and may extend larval development time, thereby increasing larval mortality due to extended exposure to predators and parasitoids [10,12]. In the case of *Y. evonymella*, however, larvae can feed and complete their development not only on native *P. padus* leaves but also on *P. serotina* with no apparent negative effects on growth and development. Leaves of the black cherry are not qualitatively worse as a food source than leaves of the bird cherry [22]. Taking into consideration evolutionary history, which predicts plant defence against a pest, we do not expect that *P. serotina* does not have pre-adapted defences [15,16]. It cannot be ruled out, however, that *P. serotina* is a pre-adapted optimal host due to the observed lack of significant differences in the defensive response among hosts [22]. As the defence and attractant signalling of *P. serotina* can be recognized as those of a host by female moths, *P. serotina* may already be considered optimal or nearly so, possibly due to a close evolutionary relationship between both shrub species (although the hosts come from different continents). Because feeding on *P. serotina* does not affect the duration of larval development [27], there is no considerable prolongation of the period that could result in a larger risk from natural enemies [10,36]. Taking the abovementioned results into consideration, the lack of difference in body mass—as well as in duration of larval development—harmonises with the fact that no significant differences in levels of parasitism among feeding variants were found in the current study. The previously observed longer period over which adults continue to emerge from pupae could, however, affect pupal-specific parasitism [22,37], although this possibility cannot yet be supported because pupal-specific parasitism has not yet been studied.

Based on the literature, the impact of alien plants on insects and their host selection preferences is well known, as can be evidenced by the large number of publications in this field [17,38,39,40,41,42,43]. In our research, we determined that the observed host expansion of *Y. evonymella* does not appear to be related to an attempt to escape from parasites, which makes alternative scenarios more likely. In situations where the optimal host is a rare species or there is heavy competition for food, the cost of feeding on the optimal host may be large enough for an insect to promote the strategy of increasing the number of potential host plants through the use of new species [4]. The inclusion of a new host plant undertaken by *Y. evonymella* may also restrain inter- and intraspecific competition [44]. *Prunus serotine,* in its European range of occurrence (in contrast to *P. padus*), is attacked by a smaller number of herbivorous species, as evidenced by a lower level of damage to its leaves [45]. Larval movement from *P. padus* (where they hatch) onto *P. serotina* during the early stages of larval development is often observed due to extensive defoliation of *P. padus* shrubs. This presents an opportunity for the moth population to avoid starvation and competition with other folivorous species. Utilising *P. serotina* may also be beneficial for *Y. evonymella* as an opportunity to improve geographical and ecological expansion, as it is known that a wider diet allows herbivores to broaden their range [46], and some herbivores benefit from the establishment and spread of exotic plants because these plants increase the amount of resources available for use [47]. In the state forests of Poland, where we first observed larval feeding on *P. serotina*, invasive black cherry is a widespread species [48]. Simultaneously, native *P. padus* is an increasingly rare species and is much more demanding in terms of soil humidity and nutrient requirements [49]. In summary, it is likely true that a noticeable abundance of *P. serotina*, in comparison to the more outcompeted main host plant *P. padus*, provides an alternative food source in the case of the total defoliation or disappearance of the primary host plant.

### 4.2. Mortality of Larvae and Structure of Parasitoid Complex

*Yponomeuta evonymella* is a species with a large range of occurrence (from Europe to East Asia), and its parasitoid complex appears to differ among geographic regions. Observations in Scandinavia have reported that *Yponomeuta* populations display regular cycles of intensification in occurrence, with strong gradations [18]. The collapse of a mass outbreak is typically caused mainly by parasitoids [50]. The transitional periods between outbreaks are characterised by approximately 50% parasitisation of the population in Central Europe [21] and approximately 30% in East Asian populations [29]. The results of our research show that a Polish population of *Y. evonymella* was in a transitional period between outbreaks, as indicated by the observed total larval parasitism (ca. 37%). The nine identified species of parasitoids found attacking our studied population of *Y. evonymella* represent ca. 15% of all species currently known to attack *Y. evonymella* [30]. 

Our study showed that, when rearing larvae on *P. padus* alone or on a mix of *P. padus* and *P. serotina*, the most important species of parasitoid are *B. aurulenta* and *B. evonymellae*. In contrast, when larvae fed on only *P. serotina*, the most common general parasitoid was *A. anxium*. 

*Agrypon anxium* (syn. *Anomalon anxium*) is a polyphagous larval/pupal parasitoid. In the past, it did not have a meaningful influence on the population of *Y. evonymella* in Poland [51] and induced parasitism rates of less than 0.5% in Asian populations [29]. In the current study, mean parasitism by this parasitoid was visibly greatest in larvae reared on *P. serotina* (more than 15-fold higher than in the *P. padus* variant). The possible reason for such a high percentage of parasitism in this variant is puzzling. As *A. anxium* is a polyphagous species, and many other species of herbivorous insects have adapted to grazing on the leaves of the alien *P. serotina* [9,52,53], it cannot be excluded that *A. anxium* is the main specialist parasitoid of any of these insect species.

*Herpestomus brunnicornis* is an endophagous parasitoid of *Yponomeuta* species, attacking mainly fifth instar, prepupal, or pupal hosts [54], although it also appears to attack the younger host stages (mid-instar host larvae) of *Y. malinellus* Zell. [37]. *Herpestomus brunnicornis* is widely distributed in Europe and causes low levels of parasitism in *Y. evonymella* [18]. In the current study, this parasitoid exhibited similar parasitism levels (ca. 2.3%) in larvae feeding on *P. padus* alone and those feeding on *P. padus*/*P. serotina*, but it was absent in larvae feeding on *P. serotina* alone. In a study [29] conducted in Korea, *H. brunnicornis* caused relatively high parasitism (6.8%) of pupal *Y. evonymella*, but it only caused 0.3% of parasitism in larvae. 

*Baryscapus evonymellae* (syn. *Tetrastichus evonymellae*) is distributed in Nearctic and Palearctic regions. It is an important endoparasitoid of *Yponomeuta* spp. [51,55] and can be called a hyperparasitoid because it is also an endoparasitoid of *D. armillata* (Hymenoptera, Ichneumonidae; [56]. 

*Diadegma armillata* is a polyphagous, abundant, and widespread endoparasitoid that is a major parasite of Yponomeutidae in Europe [18,57], although it also uses other Lepidoptera (e.g., Tortricidae, Pyralidae, and Coleophoridae). It attacks mainly larvae and occasionally pupae, and it is commonly a victim of hyperparasitism, which lowers its parasitism efficiency [56]. In related research by Lee and Pemberton [29], the levels of parasitism in *Y. evonymella* on *P. padus* were low (average 2.8% for larvae). We observed a greater share of this species in relation to total parasitism, as well as a downward trend—although insignificant—in parasitism when the food plant shifted from the original to the new host.

*Itoplectis maculator* (syn. *Ichneumonid maculator*) is a polyphagous endoparasitoid that typically attacks different small members of Lepidoptera (e.g., *Operophtera brumata* L.), Coleoptera, Hymenoptera and Diptera [58]. It is distributed across Europe and North Africa, and it has been introduced in North America [59]. In the current study, this species was not observed in the parasitoid complex of larvae that were reared on *P. serotina*.

All species in the genus *Lissonota* are oligophagous, koinobiont endoparasitoids of larval Lepidopterans [60]. These parasitoids are distributed world-wide, but the greatest diversity of *Lissonota* species occurs in the Old World, where the taxonomy of this genus has been comparatively better studied [61].

*Dirophanes invisor* (syn. *Phaenogenes invisor*; Hymenoptera; Ichneumonidae) is a polyphagous species distributed widely in Europe [62,63]. 

*Pimpla turionellae* (syn. *Ichneumon turionella*) is a polyphagous endoparasitoid, as well as a facultative hyperparasitoid [55,59,63]. It attacks individuals in the order Lepidoptera (e.g., *Y. malinellus*, *Tortrix viridana* L.) and occurs in the Palearctic. 

The tachinid *B. aurulenta* is a polyphagous, larval parasitoid that attacks many species in the genus *Yponomeuta*, but it has also been recorded on 19 other species [51]. In a Korean study, *B. aurulenta* caused low parasitism (averaging 1%) in larval *Y. evonymella* on *P. padus* [29], and, in Poland, it historically has no major impact on mortality [51]. In our study, the effect of this species in larvae reared on *P. padus* alone (ca. 6.9%) and on a mix of *P. padus*/*P. serotina* (ca. 12%) was meaningful; however, in the *P. serotina* alone variant, the effect was absent. 

### 4.3. Study Limitation

The great impact of a specific site on the level of parasitism and the species composition of parasitoids is well known [64]. The low species richness of the *Y. evonymella* parasitoids (nine species) observed in this study may be caused by the relatively small research area in an urban impact zone, as well as the fact that individuals were collected in a commercial forest stand with a simplified species structure of trees and shrubs. Despite this limitation, we believe that we correctly selected our research area because it is one of the places where this natural ecological process of host plant change from *P. padus* to *P. serotina* was first observed in *Y. evonymella*, and relatively long ago [22,27].

Furthermore, in one of the three years of previous experiments [22], we found a greater effect of parasitoids on mortality and a significant influence of the larval host plant on the level of adult eclosion, as compared to the present study. In the two remaining years of research, no effect of host species was found, and the percentage of mortality was similar to the results of the present study (approximately 40%). Transitional periods between *Y. evonymella* outbreaks are characterised by approximately 50% parasitisation of the population in Central Europe [21] and approximately 30% in East Asian populations [29], and our results do not diverge from those findings.

Finally, we would like to note that we were aware that it would be very difficult to perform an experiment to additionally capture the seasonal phenology and stage-specific parasitism of *Y. evonymellus*. We made a decision to collect all the necessary egg batches from *P. padus* at the stage before the winter diapause, into which the L1 larvae enter. The search for egg batches on *P. padus* is much simpler because there are one hundred times more eggs on its branches than on those of *P. serotina*. In the pilot study, we were able to find only three egg batches (over several days of searching), which was too small a sample size. With this study, we faced a classic trade off: We could perform an experiment by collecting egg batches from *P. padus*, but we had to remain aware that we would then not be able to understand the variability among all stages of development (and results could potentially be biased, as in the case of the egg-larval parasitoid *A. fuscicollis*). Thus, when polyembrionic *A. fuscicollis* was observed, pupae with visible marks showing emergence of this parasitoid were taken out of the container, and they were not included in this study (in the analyses).

## 5. Conclusions

In summary, changing the host plant caused differences in the structure of the parasitoid complex of *Y. evonymella* but did not improve its survival rate. This study indicates that the host expansion exhibited by *Y. evonymella* is not associated with the enemy-free space hypothesis. We identified nine species of parasitoids that attack larval *Y. evonymella* and found that the number of parasitoid species trended downwards from the primary host plant to the *P. padus*/*P. serotina* combination to the new host plant alone. We observed a significant difference among variants only in relation to the percentage of cocoons killed by specific parasitoids, but no effects of non-specific parasitoids or other factors were observed. Total mortality did not significantly differ (ca. 37%) among larval rearing variants. 

## Figures and Tables

**Table 1:** Percentage of parasitism by natural enemies reared from pupal *Yponomeuta evonymella*, which were in turn reared on *Prunus padus*, *P. serotina* or a mix of both *P. padus* and *P. serotina* shrubs (see Section 2: Materials and Methods). Means in the same row followed by different letters indicate significant differences based on Dunn’s test (*p* < 0.05). Bold values indicate *p* < 0.05. A standard deviation (SD) is given for each mean value.

Natural Enemies	Number of Parasitoids			Percentage of Parasitism (SD)			χ^2^	*p* Value
	Larval Host Plant			Larval Host Plant				
	*P. padus*	*P. padus/P. serotina*	*P. serotina*	*P. padus*	*P. padus/P. serotina*	*P. serotina*		
Diptera								
Tachinidae								
*Bactromyia aurulenta* (Meig.)	11	15	0	6.85 a (2.95)	12.20 a (5.14)	0.00 b (0.00)	8.328	**0.0155**
Hymenoptera								
Eulophidae								
*Baryscapus evonymellae* (Bché.)	29	0	3	7.21 (4.48)	0.00 (0.00)	1.19 (1.19)	5.829	0.0542
Ichneumonidae								
*Agrypon anxium* (Wesm.)	6	2	59	0.96 b (0.96)	0.28 b (0.28)	15.93 a (4.03)	23.43	**<0.0001**
*Diadegma armillata* (Grav.)	9	9	1	5.68 (4.06)	2.22 (1.24)	0.29 (0.29)	5.771	0.0558
*Herpestomus brunnicornis* Grav.	11	9	0	2.30 a (0.63)	2.35 a (1.13)	0.00 b (0.00)	8.103	**0.0174**
*Itoplectis maculator* (F.)	12	9	0	2.61 a (0.70)	2.63 a (0.90)	0.00 b (0.00)	12.58	**0.0019**
*Lissonota* sp. Grav.	6	1	2	1.29 (0.51)	0.14 (0.14)	0.46 (0.32)	4.508	0.1050
*Dirophanes invisor* (Thunb.)	2	2	0	4.30 (4.16)	0.29 (0.21)	0.00 (0.00)	2.183	0.3357
*Pimpla turionellae* L.	8	0	1	1.80 (1.13)	0.00 (0.00)	0.29 (0.29)	5.829	0.0542
Number of cocoons killed by other factors	21	43	82	4.02 (1.66)	11.56 (5.79)	20.20 (8.70)	2.578	0.2756
Total mortality				37.02 (7.01)	31.68 (6.29)	38.35 (8.01)	0.419	0.8108
Total number of killed cocoons	137	125	148					
Total number of survived cocoons	320	293	267					
Total number of moth cocoons	457	418	415					

## References

[1] Harvey J.A., Biere A., Fortuna T., Vet L.E.M., Engelkes T., Morrien E., Gols R., Verhoeven K., Vogel H., Macel M. (2010). Ecological fits, mis-fits and lotteries involving insect herbivores on the invasive plant *Bunias Orient*. Biol. Invasions.

[2] Cunningham J.P., West S.A., Zalucki M.P. (2001). Host selection in phytophagous insects: A new explanation for learning in adults. Oikos.

[3] Wiatrowska B., Łukowski A., Karolewski P., Danielewicz W. (2018). Invasive *Spiraea tomentosa*: A new host for monophagous *Earias clorana*?. Arthropod. Plant Interact..

[4] Mayhew P.J. (1997). Adaptive patterns of host-plant selection by phytophagous insects. Oikos.

[5] Dlugosch K.M., Cang F.A., Barker B.S., Andonian K., Swope S.M., Rieseberg L.H. (2015). Evolution of invasiveness through increased resource use in a vacant niche. Nat. Plants.

[6] Jeffries M.J., Lawton J.H. (1984). Enemy free space and the structure of ecological communities. Biol. J. Linn. Soc..

[7] Keane R.M., Crawley M.J. (2002). Exotic plant invasions and the enemy release hypothesis. Trends Ecol. Evol..

[8] Schönrogge K., Stone G.N., Crawley M.J., Schonrogge K. (1995). Spatial and temporal variation in guild structure: Parasitoids and inquilines of *Andricus quercuscalicis* (Hymenoptera: Cynipidae) in its native and alien ranges. Oikos.

[9] Mąderek E., Łukowski A., Giertych M.J., Karolewski P. (2015). Influence of native and alien *Prunus* species and light conditions on performance of the leaf beetle *Gonioctena quinquepunctata*. Entomol. Exp. Appl..

[10] Häggström H., Larsson S. (1995). Slow larval growth on a suboptimal willow results in high predation mortality in the leaf beetle *Galerucella lineola*. Oecologia.

[11] Williams I.S. (1999). Slow-growth, high-mortality—A general hypothesis, or is it?. Ecol. Entomol..

[12] Chen K.-W., Chen Y. (2018). Slow-growth high-mortality: A meta-analysis for insects. Insect Sci..

[13] Chupp A.D., Battaglia L.L. (2014). Potential for host shifting in *Papilio palamedes* following invasion of laurel wilt disease. Biol. Invasions.

[14] Gandhi K.J.K., Herms D.A. (2010). Direct and indirect effects of alien insect herbivores on ecological processes and interactions in forests of eastern North America. Biol. Invasions.

[15] Desurmont G.A., Donoghue M.J., Clement W.L., Agrawal A.A. (2011). Evolutionary history predicts plant defense against an invasive pest. Proc. Natl. Acad. Sci. USA.

[16] Woodard A.M., Ervin G.N., Marsico T.D. (2012). Host plant defense signaling in response to a coevolved herbivore combats introduced herbivore attack. Ecol. Evol..

[17] Burghardt K.T., Tallamy D.W., Philips C., Shropshire K.J. (2010). Non-native plants reduce abundance, richness, and host specialization in lepidopteran communities. Ecosphere.

[18] Junnikkala E. (1960). Life history and insect enemies of *Hyponomeuta malinellus* Zell. (Lep., Hyponomeutidae) in Finland. Ann. Zool. Soc. Zool. Fenn. Vanamo.

[19] Leather S.R. (1986). Insects on bird cherry I. The bird cherry ermine moth, *Yponomeuta evonymellus* (L.) (Lepidoptera: Yponomeutidae). Entomol. Gaz..

[20] Menken S.B.J., Herrebout W.M., Wiebes J.T. (1992). Small ermine moths (*Yponomeuta*): Their host relations and evolution. Annu. Rev. Entomol..

[21] Alonso C., Vuorisalo T., Wilsey B., Honkanen T. (2000). *Yponomeuta evonymellus* outbreaks in southern Finland: Spatial synchrony but different local magnitudes. Ann. Zool. Fenn..

[22] Karolewski P., Jagodziński A.M., Giertych M.J., Łukowski A., Baraniak E., Oleksyn J. (2014). Invasive *Prunus serotine*—A new host for *Yponomeuta evonymellus* (Lepidoptera: Yponomeutidae)?. Eur. J. Entomol..

[23] Karolewski P., Łukowski A., Walczak U., Baraniak E., Mucha J., Giertych M.J. (2017). Larval food affects oviposition preference, female fecundity and offspring survival in *Yponomeuta evonymellus*. Ecol. Entomol..

[24] Pairon M., Petitpierre B., Campbell M., Guisan A., Broennimann O., Baret P.V., Jacquemart A.L., Besnard G. (2010). Multiple introductions boosted genetic diversity in the invasive range of black cherry (*Prunus serotina*; Rosaceae). Ann. Bot..

[25] Houston Durrant T., Caudullo G., San-Miguel-Ayanz J., de Rigo D., Caudullo G., Houston Durrant T., Mauri A. (2016). *Prunus padus* in Europe: Distribution, habitat, usage and threats. European Atlas of Forest Tree Species.

[26] Łukowski A., Mąderek E., Karolewski P. (2014). Light conditions effect on bird cherry ermine moth—The main pest of bird cherry. Sylwan.

[27] Łukowski A., Giertych M.J., Walczak U., Baraniak E., Karolewski P. (2017). Light conditions affect the performance of *Yponomeuta evonymellus* on its native host *Prunus padus* and the alien *Prunus serotina*. Bull. Entomol. Res..

[28] Dixon A.F.G. (1971). The life-cycle and host preferences of the bird cherry-oat aphid, *Rhopalosiphum padi* L., and their bearing on the theories of host alternation in aphids. Ann. Appl. Biol..

[29] Lee J.-H., Pemberton R.W. (2009). Parasitoid complex of the bird cherry ermine moth *Yponomeuta evonymellus* in Korea. Entomol. Res..

[30] Žikić V., Lotfalizadeh H., Schwarz M., Stanković S.S., Lazarević M., Kos K., Rakhshani E., Tschorsnig H.-P. (2018). Parasitoids of European species of the genus *Yponomeuta* Latreille 1796 (Lepidoptera: Yponomeutidae): New findings with an updated checklist. Phytoparasitica.

[31] Vanhellemont M., Wauters L., Baeten L., Bijlsma R.-J., De Frenne P., Hermy M., Verheyen K. (2010). *Prunus serotina* unleashed: Invader dominance after 70 years of forest development. Biol. Invasions.

[32] Ehrlich P.R., Raven P.H. (1964). Butterflies and plants: A study in coevolution. Evolution.

[33] Coley P.D., Bateman M.L., Kusar T.A. (2006). The effects of plant quality on caterpillar growth and defense against natural enemies. Oikos.

[34] Awmack C., Leather S. (2002). Host plant quality and fecundity in herbivorous insects. Annu. Rev. Entomol..

[35] Walczak U., Baraniak E., Zduniak P. (2017). Survival, body mass and potential fecundity of the invasive moth *Cameraria ohridella* (Lepidoptera: Gracillariidae) on its original host plant *Aesculus hippocastanum* and *Aesculus glabra*. Eur. J. Entomol..

[36] Fortuna T.M., Woelke J.B., Hordijk C.A., Jansen J.J., van Dam N.M., Vet L.E.M., Harvey J.A. (2013). A tritrophic approach to the preference-performance hypothesis involving an exotic and a native plant. Biol. Invasions.

[37] Lee J.-H., Pemberton R.W. (2007). Seasonal phenology and stage-specific parasitism of the apple ermine moth, *Yponomeuta malinellus* Zeller, in Korea. Entomol. Res..

[38] Burghardt K.T., Tallamy D.W., Gregory Shriver W. (2009). Impact of native plants on bird and butterfly biodiversity in suburban landscapes. Conserv. Biol..

[39] Tallamy D.W., Ballard M., D’Amico V. (2010). Can alien plants support generalist insect herbivores?. Biol. Invasions.

[40] Narango D.L., Tallamy D.W., Marra P.P. (2017). Native plants improve breeding and foraging habitat for an insectivorous bird. Biol. Conserv..

[41] Narango D.L., Tallamy D.W., Marra P.P. (2018). Nonnative plants reduce population growth of an insectivorous bird. Proc. Natl. Acad. Sci. USA.

[42] Richard M., Tallamy D.W., Mitchell A.B. (2019). Introduced plants reduce species interactions. Biol. Invasions.

[43] Tallamy D.W., Shropshire K.J. (2009). Ranking lepidopteran use of native versus introduced plants. Conserv. Biol..

[44] Feder J.L., Reynolds K., Go W., Wang E.C. (1995). Intra- and interspecific competition and host race formation in the apple maggot fly, *Rhagoletis pomonella* (Diptera: Tephritidae). Oecologia.

[45] Karolewski P., Giertych M.J., Żmuda M., Jagodziński A.M., Oleksyn J. (2013). Season and light affect constitutive defenses of understory shrub species against folivorous insects. Acta Oecol..

[46] Keeler M.S., Chew F.S. (2008). Escaping an evolutionary trap: Preference and performance of a native insect on an exotic invasive host. Oecologia.

[47] Harvey J.A., Fortuna T.M. (2012). Chemical and structural effects of invasive plants on herbivore-parasitoid/predator interactions in native communities. Entomol. Exp. Appl..

[48] Bijak S., Czajkowski M., Ludwisiak Ł. (2015). Occurrence of *Black cherry* (*Prunus serotina* Ehrh.) in the State Forests in Poland. For. Res. Pap..

[49] Ellenberg H., Weber H., Düll R., Wirth V., Werner W., Paulißen D. (1992). Zeigerwerte von Pflanzen in Mitteleuropa. Scr. Geobot..

[50] Pyörnilä M., Pyörnilä A. (1979). Role of parasitoids in termination of a mass occurrence of *Yponomeuta evonymellus* (Lepidoptera, Yponomeutldae) in northern Finland. Not. Entomol..

[51] Karczewski J. (1980). Przyczynek do poznania entomofagów namiotnika czaremszaczka (*Hyponomeuta evonymellus* L.) (Lep. Hyponomeutodae). Sylwan.

[52] Kuhlmann U., Babendreier D., Hoffmeister T.S., Mills N.J. (1998). Impact and oviposition behaviour of *Ageniaspis fuscicollis* (Hymenoptera: Encyrtidae), a polyembryonic parasitoid of the apple ermine moth, *Yponomeuta malinellus* (Lepidoptera: Yponomeutidae). Bull. Entomol. Res..

[53] Cossentine J.E., Kuhlmann U. (2000). Status of *Ageniaspis fuscicollis* (Hymenoptera: Encrytidae), an introduced parasitoid of the *Apple ermine moth* (Lepidoptera: Yponomeutidae). Can. Entomol..

[54] Dijkerman H.J. (1987). Parasitoid complexes and patterns of parasitization in the genus *Yponomeuta* Latreille (Lepidoptera, Yponomeutidae). J. Appl. Entomol..

[55] Nowakowska K., Halarewicz A. (2006). Coleoptera found on neophyte *Prunus serotina* (Ehrh.) within forest community and open habitat. Electron. J. Polish Agric. Univ..

[56] Nowakowska K., Halarewicz A. (2006). *Prunus serotina* (Ehrh.)—New food resource for polyphagous Lepidoptera. Electron. J. Polish Agric. Univ. Bilology.

[57] Unruh T., Short R., Herard F., Chen K., Hopper K., Pemberton R., Hoon Lee J., Ertle L., Swan K., Fuester R. (2003). Introduction and establishment of parasitoids for the biological control of the apple ermine moth, *Yponomeuta malinellus* (Lepidoptera: Yponomeutidae), in the Pacific Northwest. Biol. Control..

[58] Menken S. (1982). Enzymatic characterization of nine endoparasite species of small ermine moths (Yponomeutidae). Experientia.

[59] Yefremova Z., Ebrahim E., Yegorenkova E. (2007). The subfamilies Eulophinae, Entedoninae and Tetrastichinae in Iran, with description of new species (Hymenoptera: Eulophidae). Entomofauna.

[60] Dijkerman H.J. (1990). Suitability of eight *Yponomeuta*-species as hosts of *Diadegma armillata*. Entomol. Exp. Appl..

[61] Shaw M.R. (2009). Notes on the host-feeding and hyperparasitic behaviours of *Itoplectis* species (Hymenoptera: Ichneumonidae, Pimplinae). Entomol. Gaz..

[62] Kolarov J.A., Gürbuz M.F. (2004). A study of the Turkish *Ichneumonidae* (Hymenoptera). I. Pimpilinae. Linzer Biol. Beiträge.

[63] Çoruh S., Özbek H. (2013). New and little known some *Ichneumonidae* species (Hymenoptera) from Turkey. Munis Entomol. Zool..

[64] Amiri A., Talebi A.A., Rakhshani E., Hajiqanbar H. (2016). Study of the genus *Lissonota* (Hymenoptera: Ichneumonidae: Banchinae) in southern Iran. J. Entomol. Soc. Iran..

